# SAMHD1 expression contributes to doxorubicin resistance and predicts survival outcomes in diffuse large B-cell lymphoma patients

**DOI:** 10.1093/narcan/zcae007

**Published:** 2024-02-24

**Authors:** Waaqo Daddacha, Dominique Monroe, Ashley J Schlafstein, Allison E Withers, Elizabeth B Thompson, Diana Danelia, Nho C Luong, Fatmata Sesay, Sandip K Rath, Edidiong R Usoro, Mark E Essien, Andrew T Jung, Jinmeng G Jiang, Jiaxuan Hu, Bijan Mahboubi, Arilyn Williams, Julia E Steinbeck, Xiaofeng Yang, Zachary S Buchwald, William S Dynan, Jeffrey M Switchenko, Baek Kim, Mohammad K Khan, David L Jaye, David S Yu

**Affiliations:** Department of Biochemistry and Molecular Biology, Medical College of Georgia, Augusta University, Augusta, GA 30912, USA; Department of Biochemistry and Molecular Biology, Medical College of Georgia, Augusta University, Augusta, GA 30912, USA; Department of Radiation Oncology, Emory University School of Medicine, Atlanta, GA 30322, USA; Department of Radiation Oncology, Emory University School of Medicine, Atlanta, GA 30322, USA; Department of Radiation Oncology, Emory University School of Medicine, Atlanta, GA 30322, USA; Department of Radiation Oncology, Emory University School of Medicine, Atlanta, GA 30322, USA; Department of Radiation Oncology, Emory University School of Medicine, Atlanta, GA 30322, USA; Department of Radiation Oncology, Emory University School of Medicine, Atlanta, GA 30322, USA; Department of Radiation Oncology, Emory University School of Medicine, Atlanta, GA 30322, USA; Department of Biochemistry and Molecular Biology, Medical College of Georgia, Augusta University, Augusta, GA 30912, USA; Department of Radiation Oncology, Emory University School of Medicine, Atlanta, GA 30322, USA; Department of Radiation Oncology, Emory University School of Medicine, Atlanta, GA 30322, USA; Department of Radiation Oncology, Emory University School of Medicine, Atlanta, GA 30322, USA; Department of Radiation Oncology, Emory University School of Medicine, Atlanta, GA 30322, USA; Department of Pediatrics, Emory University School of Medicine, Atlanta, GA 30322, USA; Department of Biochemistry and Molecular Biology, Medical College of Georgia, Augusta University, Augusta, GA 30912, USA; Department of Biochemistry and Molecular Biology, Medical College of Georgia, Augusta University, Augusta, GA 30912, USA; Department of Radiation Oncology, Emory University School of Medicine, Atlanta, GA 30322, USA; Department of Radiation Oncology, Emory University School of Medicine, Atlanta, GA 30322, USA; Department of Radiation Oncology, Emory University School of Medicine, Atlanta, GA 30322, USA; Department of Biochemistry, Emory University School of Medicine, Atlanta, GA 30322, USA; Department of Biostatistics and Bioinformatics, Rollins School of Public Health and Winship Cancer Institute, Emory University School of Medicine, Atlanta, GA 30322, USA; Department of Pediatrics, Emory University School of Medicine, Atlanta, GA 30322, USA; Department of Radiation Oncology, Emory University School of Medicine, Atlanta, GA 30322, USA; Department of Pathology and Laboratory Medicine, Emory University School of Medicine, Atlanta, GA 30322, USA; Department of Radiation Oncology, Emory University School of Medicine, Atlanta, GA 30322, USA

## Abstract

Diffuse large B-cell lymphoma (DLBCL) is a commonly diagnosed, aggressive non-Hodgkin's lymphoma. While R-CHOP chemoimmunotherapy is potentially curative, about 40% of DLBCL patients will fail, highlighting the need to identify biomarkers to optimize management. SAMHD1 has a dNTPase-independent role in promoting resection to facilitate DNA double-strand break (DSB) repair by homologous recombination. We evaluated the relationship of SAMHD1 levels with sensitivity to DSB-sensitizing agents in DLBCL cells and the association of SAMHD1 expression with clinical outcomes in 79 DLBCL patients treated with definitive therapy and an independent cohort dataset of 234 DLBCL patients. Low SAMHD1 expression, Vpx-mediated, or siRNA-mediated degradation/depletion in DLBCL cells was associated with greater sensitivity to doxorubicin and PARP inhibitors. On Kaplan–Meier log-rank survival analysis, low SAMHD1 expression was associated with improved overall survival (OS), which on subset analysis remained significant only in patients with advanced stage (III-IV) and moderate to high risk (2–5 International Prognostic Index (IPI)). The association of low SAMHD1 expression with improved OS remained significant on multivariate analysis independent of other adverse factors, including IPI, and was validated in an independent cohort. Our findings suggest that SAMHD1 expression mediates doxorubicin resistance and may be an important prognostic biomarker in advanced, higher-risk DLBCL patients.

## Graphical Abstract

**Graphical Abstract:**
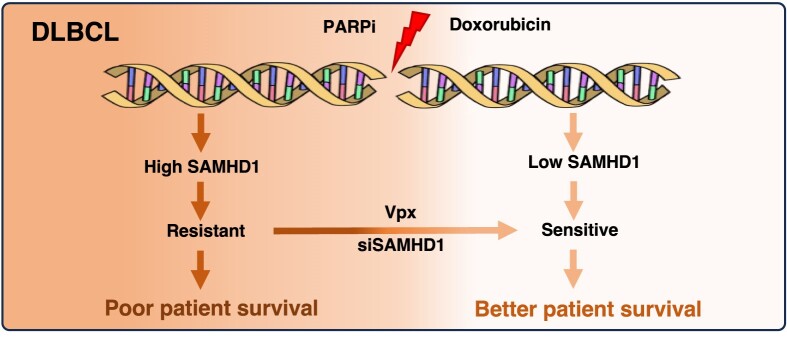

## Introduction

Diffuse large B-cell lymphoma (DLBCL) is the most commonly diagnosed non-Hodgkin's lymphoma, accounting for up to 25% of adult lymphoma diagnoses (1,2). Generally, DLBCLs are categorized into germinal center B-cell-like (GCB) and active B-cell-like (ABC) subtypes based on their cell of origin (3,4). However, uncategorized subtypes account for about 15% of DLBCL. Although several studies have shown that patients with GCB DLBCL subtype have a better prognosis than ABC, it was also demonstrated that subtypes and their response to therapy are heterogeneous, with variable outcomes. Indeed, studies have shown that DLBCL subtypes differ in genetic makeup, clinical presentation, and response to therapy (5–8).

Mutations and dysregulation of a sterile alpha motif (SAM) and histidine-aspartic acid (HD) domain-containing protein 1 (SAMHD1) are associated with Aicardi Goutières syndrome (AGS) (9), an inherited autoimmune encephalopathic disorder, and several cancers (10), including leukemias (11–13), lymphomas (14–16), colorectal cancer (17), and lung cancer (18). SAMHD1 has a deoxyribonucleoside (dNTP) triphosphohydrolase activity (19,20) essential for clinical response to dNTP analog therapeutic agents, including cytarabine and decitabine in acute myeloid leukemia (AML) (21–24). SAMHD1 also has a dNTPase-independent role in the DNA damage response (DDR) by promoting DNA end resection to facilitate DNA double-strand break (DSB) repair by homologous recombination (HR) (25,26) and resection of nascent DNA at stalled replication forks (27). The end resection function of SAMHD1 is regulated by deacetylation at lysine 354 (K354) by the sirtuin 1 (SIRT1) deacetylase, which facilitates its binding to ssDNA at DSBs (28). Given its role in DSB repair, SAMHD1 may be an essential biomarker of resistance and clinical outcomes to DSB-inducing therapeutic agents. One of these agents is doxorubicin, an intercalating agent that targets cellular DNA to inhibit topoisomerase II function, leading to DSBs (29,30) and a critical component of DLBCL therapy.

DLBCL is primarily treated with R-CHOP, a standard combined chemoimmunotherapeutic regimen of rituximab, cyclophosphamide, doxorubicin, vincristine, and prednisone (1,2). While the overall survival (OS) rate of DLBCL patients is about a year without treatment, R-CHOP therapy achieves a 5-year OS rate of 60–70% (31,32). However, patients who do not achieve complete response or relapse after R-CHOP therapy have been reported to have worse outcomes (33–36). In recent years, poly ADP-ribose polymerase (PARP) inhibitors have been explored as a potential therapeutic approach for DLBCL in preclinical models (37) and in DLBCL patients in the relapsed or refractory setting (38). The International Prognostic Index (IPI) scoring system for DLBCL predicts outcomes based on several risk factors, including age >60, stage III/IV, Eastern Cooperative Oncology Group (ECOG) performance status ≥2, serum LDH level above the upper limit of normal, and extranodal site involvement > 1 (39); however, even amongst IPI risk groups, there are variable outcomes. Thus, there is a need for prognostic biomarkers to better stratify patients and assist in selecting therapeutic strategies. Here, we show that low SAMHD1 expression or degradation/depletion is associated with greater sensitivity to clinically relevant DSB-inducing agents’ doxorubicin and PARP inhibitor in DLBCL cells and that low SAMHD1 expression is associated with improved OS in DLBCL patients independent of other adverse factors, including IPI, and is most significant for those with advanced stage and higher risk.

## Materials and methods

### Cell culture and western blot

SUDHL-4, SUDHL-6, RCK-8, and RIVA cell lines were grown in Roswell Park Memorial Institute (RPMI) Media 1640 (GIBCO, 11875-093) supplemented with 20% fetal bovine serum (FBS, Atlanta Biologicals, S1150) as described before (40). The cell lines were grown in flasks in an incubator (37°C, 5% carbon dioxide). Cell lines described above were harvested and lysed for 30 min on ice in radioimmunoprecipitation assay (RIPA) buffer supplemented with protease inhibitors. Lysates underwent centrifugation at max speed for 10 min at 4°C, and the supernatants were collected. Protein concentration for each sample was measured using the Bradford assay. 30 μg of total protein was resolved by SDS-PAGE and visualized with LI-COR Odyssey observer after staining with primary antibodies (SAMHD1 (OriGene, TA502024) and GAPDH (Santa Cruz, sc-47724) followed by the probe with Alexa Fluor anti-mouse or anti-rabbit secondary antibodies (Life Technologies).

### Cell sensitivity and apoptosis assay

Cell sensitivity assays were performed via AlamarBlue cell viability assay as previously described (41,42) or propidium iodide (PI)/Annexin V-based flow cytometry assay. Briefly, cells were plated at a density of 1 × 10^6^ cells/ml in a 25ml cell culture flask and transduced with VLPs with or without Vpx as described previously (43,44). The cells were incubated with a medium containing VLPs for 48 h, counted, and re-plated in triplicate to a density of 1 × 10^4^ cells/well in 96-well plates. 24 h after plating, cells were treated with the drug (doxorubicin or veliparib) for 72 h. Cell viability was assessed via AlamarBlue (Resazurin) reagent, incubated at 1:10 dilution for 6 h, and assayed for fluorescence according to the manufacturer's protocol. Viability fractions were normalized to mock-treated controls exposed to identical conditions. Evaluation of cell viability using Annexin V and Propidium Iodide (FITC/PI) staining following treatment of cells with 0, 2, 4, 8 and 16 uM veliparib or 0, 75, 150, 300 nM Doxorubicin. Graphs show the average percentage of viable cells after treatment with Veliparib or Doxorubicin as determined by flow cytometry. 500K cells per well were plated in a 6-well plate and treated with an indicated dose of Veliparib or Doxorubicin for 48 h, followed by flow cytometry. The live cells were determined, and data was presented as the mean ± standard deviation of three independent experiments. For the siRNA-mediated SAMHD1 KD experiments, cells were transfected with siNT and siSAMHD1 24 h before Doxorubicin treatment. For Vpx-mediated SAMHD1 degradation, cells were transduced with VLP (+Vpx) 24 h prior to treatment with doxorubicin. For analysis of apoptosis, cells were analyzed by flow cytometry using Annexin V/7-AAD following treatment.

### Cell cycle analysis and DNA damage

RCK-8 and SUDHL-6 cells transduced with VLPs (–/+ Vpx) were harvested, washed with PBS, and fixed in 70% ice-cold ethanol. The fixed cells were washed in BPS and stained with 25 μg/ml propidium iodide (Sigma)/ 10 μg/ml RNase (Qiagen). Fixed cells were sorted on a FACS Canto II (BD Bioscience) and analyzed with FlowJo software. To assess DNA damage repair, SUDHL-4 cells were incubated and transduced with VLPs containing Vpx. The cells were incubated for 24 h and treated with 250 nM Doxorubicin for 48 h. Then, cells were harvested and followed by western blot analysis as described above. The membrane was probed with anti-SAMHD1 antibody to confirm degradation and with anti-RPA32 to assess DNA damage. RPA32 gel shift was monitored to determine its phosphorylation.

### dNTP assay

RCK-8 and SUDHL-6 cellular dNTP pools were measured as described previously (45,46). Briefly, cells were depleted of SAMHD1 by transducing with VLPs containing Vpx. Cells were washed with PBS, lysed in 65% aqueous methanol, and then heated at 95°C. Lysed samples were centrifuged at maximum speed for 10 min, dried, and resuspended in water. The cell extracts were used as a dNTP source to extend a single nucleotide at the 5′ end of the 18-nucleotide primer annealed to 19-nucleotide templates with varying overhangs (A, T, C or G). The products were resolved on urea-PAGE, and the amount of extended /unextended primers was quantified with QuantityOne software. Dilutions required to obtain extension within the linear range were determined for each sample and used to perform the assay.

### Patient selection

Patients diagnosed with DLBCL were selected from Emory University's electronic medical record system (EMR) between January 1991 and August 2009. Overall survival (OS) was calculated from the date of diagnosis to the date of the last contact as indicated in the EMR or date of death. Patient demographics, treatment category, and timeline of patient contact in the Emory clinical system were determined by chart review through the EMR. Emory Institutional Review Board (IRB) obtained and kept patient consent on file. Patient privacy and confidentiality were preserved per the Health Insurance Portability and Accountability Act of 1996 (HIPAA).

### Genomics data commons

Log_2_ trimmed mean of M values (TMM) normalized SAMHD1 expression data (*n* = 562) along with the clinical information was extracted from three cancer programs within the Genomics Data Commons (GDC) data portal (47). The data contains 40 cases from The Cancer Genome Atlas (TCGA), 41 cases from the Clinical Trial Sequencing Project (CTSP), and 481 cases from the NCI Center for Cancer Research (NCICCR). While the dataset contained 574 cases, only the NCICCR had data available for the survival analysis. SAMHD1 was dichotomized at the median log_2_ normalized value into low/high. The data have SAMHD1 expression values between 4.6 and 9.6. As a result, we split the data using its median value (7.18) and compared the SAMHD1 low (less than the median) and SAMHD1 high (more significant than the median) groups. We censored observations with more than five years of follow-up time, and Kaplan–Meier plots reported overall survival. The SAMHD1 mRNA expression distributions for the NCICCR dataset are presented (Supplementary Figure S3E).

### Immunohistochemical analysis

Tissue microarrays (TMAs) were constructed (3 cores/case, 1 mm diameter) from archived formalin-fixed, paraffin-embedded (FFPE) tissue sections from biopsies of 89 patient cases of DLBCL patients treated at Emory University Hospital for which treatment, survival, staging, and demographic data were available. Appropriate institutional (IRB) approvals were obtained. Diagnoses rested on the World Health Organization (WHO) classification (48). Cases were excluded from final analyses if they lacked evaluable cores (no tissue or DLBCL). TMA sections underwent processing and immunostaining on a DAKO autostainer as described previously (49). All TMAs were stained with H&E, CD3 for T cells, and CD20 and Pax5 for B cells. The combination of overall tissue architecture, lymphoma cytomorphology, and immunostaining results across these stains, as determined with manual microscopy, is a traditional method for identifying large B cell lymphoma cells and distinction from non-neoplastic reactive background cells and was employed here. Anti-SAMHD1 mouse monoclonal antibody (Clone ID: OTI3F5, ORIGENE) was used at 1:640. The specificity of the anti-SAMHD1 antibody was validated by western blot analysis following SAMHD1 degradation. Positivity for SAMHD1 required any lymphoma cell nuclear reactivity with negative background cells and was averaged across all available cores per case. The percentage of immunoreactive lymphoma cell nuclei for SAMHD1 was averaged across the tissue cores for each case. A score of >3 is defined as greater than the median amongst the cases for the percent of lymphoma cell nuclei that were immunoreactive for SAMHD1. Antibodies to CD3, CD10, CD20, BCL6 and MUM1 were used for the cell of origin determination by the Hans algorithm (50). Immunostaining for MYC and BCL2 was performed to determine the double expression status as described. MYC/BLC2 double expressing was defined as ≥40% MYC and ≥50% BCL2. Immunohistochemical double-hit score is a strong predictor of outcomes in DLBCL patients treated with rituximab plus cyclophosphamide, doxorubicin, vincristine, and prednisone (51). All TMAs were scored by the experienced hematologist blinded to clinical outcomes and other clinical data.

### Statistical analysis

Overall survival (OS) was defined as the time from diagnosis to death or the last contact when living patients were censored at the last contact. OS was estimated using the Kaplan–Meier method and was compared using log-rank tests. 1-year, 2-year and 5-year survival estimates were reported, along with median survival. Cut points for SAMHD1 included the median (3), 14, and 25. Univariate Cox proportional hazards models were fit for SAMHD1 cut points, as well as race (white vs. non-white), sex (male vs. female), stage (I/II versus III/IV), treatment (R-CHOP versus others), LDH (≤271 versus >271), IPI score (0–1 versus 2–5), and age at diagnosis (both continuous and ≤60 versus >60. Multivariable Cox models were fit for the median SAMHD1 cut point, controlling for stage, age and other statistically significant variables on univariate analysis. Multivariable Cox models were also fit for the median SAMHD1 cut point (>3) controlling for IPI score alone. Statistical analyses were performed using SAS 9.4 (SAS Institute Inc., Cary, NC), and statistical significance was assessed at the 0.05 level. To assess for SAMHD1 correlation with MYC and BCL2, percent high SAMHD1, defined as a value greater than 3, was compared between those with and without double expressors, defined as ≥40% MYC and ≥50% BCL2, using a Chi-square test. Two-tailed *t*-test analysis was utilized to determine the statistical difference in all cell viability assays.

## Results

### Low SAMHD1 expression is associated with greater sensitivity to doxorubicin and PARP inhibitors in DLBCL

**Figure 1.**
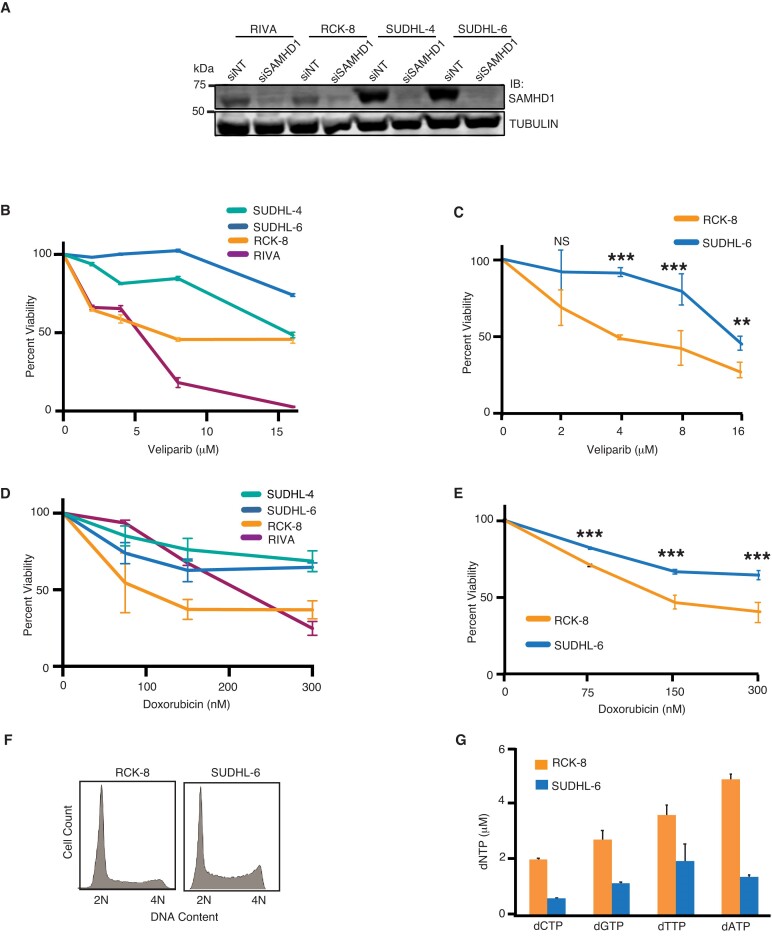
Low SAMHD1 expression is associated with greater sensitivity to doxorubicin and Veliparib in DLBCL. (**A**) DLBCL cell lines RCK-8, RIVA, SUDHL-4 and SUDHL-6 were transfected with siSAMHD1, lysed, run on SDS-PAGE, and analyzed for SAMHD1 expression by western blot analysis. (B, C) Indicated cells were seeded and treated with varying doses of Veliparib, and viability was analyzed via PI/Annexin V assay (**B**) or AlamarBlue assay (**C**). (D, E) Indicated cells were seeded and incubated with varying concentrations of doxorubicin, and viability was analyzed via PI/Annexin V assay (**D**) or AlamarBlue assay (**E**). (**F**) RCK8 and SUDHL-6 were fixed, PI stained, and cell cycle was analyzed by flow cytometry. (**G**) RCK-8 and SUDHL-6 were lysed, dNTP extracted, and RT-based primer extension assay was analyzed for dNTP pool size. For B, C, D, E and G, the mean and standard deviation from three replicas are shown. ** *P* < 0.01, *** *P* < 0.001.

To determine if SAMHD1 is differentially expressed in DLBCL cells, we analyzed a panel of DLBCL cell lines: RCK-8, RIVA, SUDHL-4 and SUDHL-6. SAMHD1 was expressed at higher levels in SUDHL-4 and -6 cells than in RCK-8 and RIVA cells (Figure 1A), suggesting that SAMHD1 is differentially expressed in DLBCL cells. We confirmed that the band observed was SAMHD1 by depleting SAMHD1 using siRNA. Cancer cell sensitivity to DNA damage-inducing agents has been extensively utilized to determine the role of genes in the DNA damage response (DDR). Dysregulation of genes involved in HR often confers greater sensitivity to PARP inhibitors. Indeed, we have shown that SAMHD1 depletion or degradation in cancer cells enhances sensitivity to Veliparib, a PARP inhibitor (25,26). To determine whether SAMHD1 levels influence DLBCL response to PARP inhibitors, we examined cellular sensitivity to Veliparib in RCK-8, RIVA, SUDHL-4 and SUDHL-6 cell lines with lower and higher SAMHD1 levels, as shown above. RCK-8 and RIVA, DLBCL cell lines with lower SAMHD1 expression showed, in general, greater sensitivity to Veliparib as compared to SUDHL-4 and SUDHL-6, DLBCL cell lines with higher SAMHD1 expression as determined with a PI/Annexin V assay where live cells were considered (Figure 1B and Supplementary Figure S1A). This observation was confirmed with an AlamarBlue cell viability assay by comparing RCK-8 and SUDHL-6 (Figure 1C), suggesting that low SAMHD1 expression is associated with DLBCL cell Veliparib greater sensitivity and consistent with a role for SAMHD1 in promoting HR. Furthermore, we examined cellular sensitivity in these cells to determine whether DLBCL cell lines have differential sensitivity to doxorubicin, a significant component of R-CHOP therapy that is known to induce DSBs (52,53). Consistently, RCK-8 and RIVA cells with lower SAMHD1 expression demonstrated in general greater doxorubicin sensitivity compared with SUDHL-4 and SUDHL-6 cells as determined via a PI/Annexin V assay (Figure 1D and Supplementary Figure S1B) and AlamarBlue cell viability assay (Figure 1E), indicating a potential SAMHD1 contribution in repairing doxorubicin-induced DNA damage. Only a modest difference in cellular dNTP pools was observed between RCK-8 and SUDHL-6 cells (Figure 1G), while SUDHL-6 cells had a greater proportion of cells in S and G2/M phases (Figure 1F). These data suggest that SAMHD1 expression levels could determine DLBCL resistance to DSB-inducing therapeutic agents.

### SAMHD1 depletion and degradation causes doxorubicin sensitization

**Figure 2.**
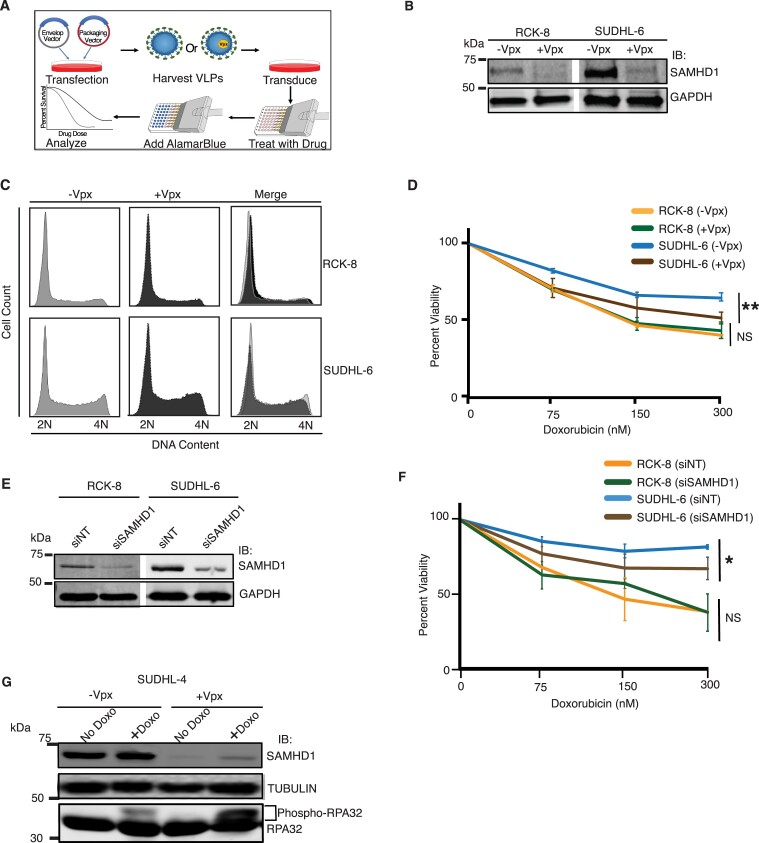
SAMHD1 degradation with VLPs with Vpx causes doxorubicin sensitization. (**A**) Schematic of VLP preparation and viability assay. (**B**) Western blot analysis showing Vpx-mediated degradation of SAMHD1 in RCK-8 and SUDHL-6 cells. (**C**) RCK-8 and SUDHL-6 were cultured in fresh media at sub-confluency and transduced with VLPs with or without Vpx. 48 h post-transduction, cells were fixed, stained with PI, and analyzed for cell cycle profile by flow cytometry. (**D**) 48 h post-transduction with VLPs with Vpx (+Vpx) or without Vpx (-Vpx), RCK-8 and SUDHL-6 cells were seeded in 96-well plates, incubated with indicated concentrations of doxorubicin for 48 hr, and viability was determined by assessing metabolization of AlamarBlue. (**E**) Western blot analysis showing siRNA-mediated SAMHD1 depletion in RCK-8 and SUDHL-6 cells. (**F**) Evaluation of cell viability using PI/Annexin V staining following siRNA-mediated SAMHD1 knockdown following treatment with varying concentrations of Doxorubicin for 48 h. (**G**) SUDHL-4 cells were transduced with VLPs without Vpx (-Vpx) or with Vpx (+Vpx) and incubated for 24 h and treated with 250 nM doxorubicin for 48 h. Then, cells were harvested, lysed, resolved on SDS gel, and probed for indicated proteins. For D and E, mean and standard deviation obtained from three replicas is shown. ** *P* < 0.01 and * *P* < 0.05

While the data presented above suggest a correlation between SAMHD1 expression levels and resistance to DSB-sensitizing agents in DLBCL, a causal effect requires isogenic conditions. However, DLBCL cells are difficult to transfect, making utilization of siRNA for gene depletion challenging. It is well established that SAMHD1 is targeted for proteasomal degradation by viral protein X (Vpx), a protein encoded by human immunodeficiency virus 2 (HIV-2) and simian immunodeficiency virus (SIV) (43,54,55). Furthermore, Vpx can be packaged into vesicular stomatitis virus glycoprotein (VSV-G) envelope-coated virus-like particles (VLPs) and delivered into a target cell (26,54,56). We exploited this strategy to successfully degrade SAMHD1 in RCK-8 and SUDHL-6 cells (Figure 2A, B). There was no significant change in cell cycle observed in SUDHL-6 and RCK-8 cells following transduction with VLPs containing Vpx (Figure 2C). Interestingly, Vpx-mediated SAMHD1 degradation caused a significant increase in sensitization to doxorubicin in SUDHL-6 cells with higher SAMHD1 expression, in contrast to RCK-8 cells which exhibit lower SAMHD1 expression as determined by AlamarBlue based cell viability assay (Figure 2D). This suggests that SAMHD1 contributes to doxorubicin resistance when expressed at higher levels and that resistant SUDHL-6 cells can be sensitized to doxorubicin by Vpx-mediated SAMHD1 degradation. Greater sensitivity of SUDHL-6 cells to doxorubicin was confirmed by PI/Annexin V assay following siRNA-mediated SAMHD1 depletion (Figure 2E-F, and Supplementary Figure S2A–C). Similarly, SUDHL-4, another DLBCL cell line with higher SAMHD1 expression, was sensitized to doxorubicin treatment following Vpx-mediated SAMHD1 degradation and siRNA-mediated SAMHD1 depletion (Supplementary Figure S2D-F). Higher SAMHD1 expressing DLBCL cells’ sensitivity to doxorubicin following siRNA-mediated SAMHD1 degradation was confirmed by an AlamarBlue-based cell viability assay (Supplementary Figure S2G–I). Furthermore, Vpx-mediated SAMHD1 degradation resulted in increased phospho-RPA32 as determined by a mobility shift of RPA32 following doxorubicin treatment (Figure 2G), suggesting that SAMHD1 degradation increases doxorubicin-induced DNA damage. Collectively, these data indicate that SAMHD1 depletion and degradation causes greater sensitivity to doxorubicin treatment, demonstrating the potential of targeting SAMHD1 with VLPs containing Vpx to sensitize DLBL cells to doxorubicin.

### Low SAMHD1 expression is associated with improved overall survival in DLBCL patients

**Figure 3.**
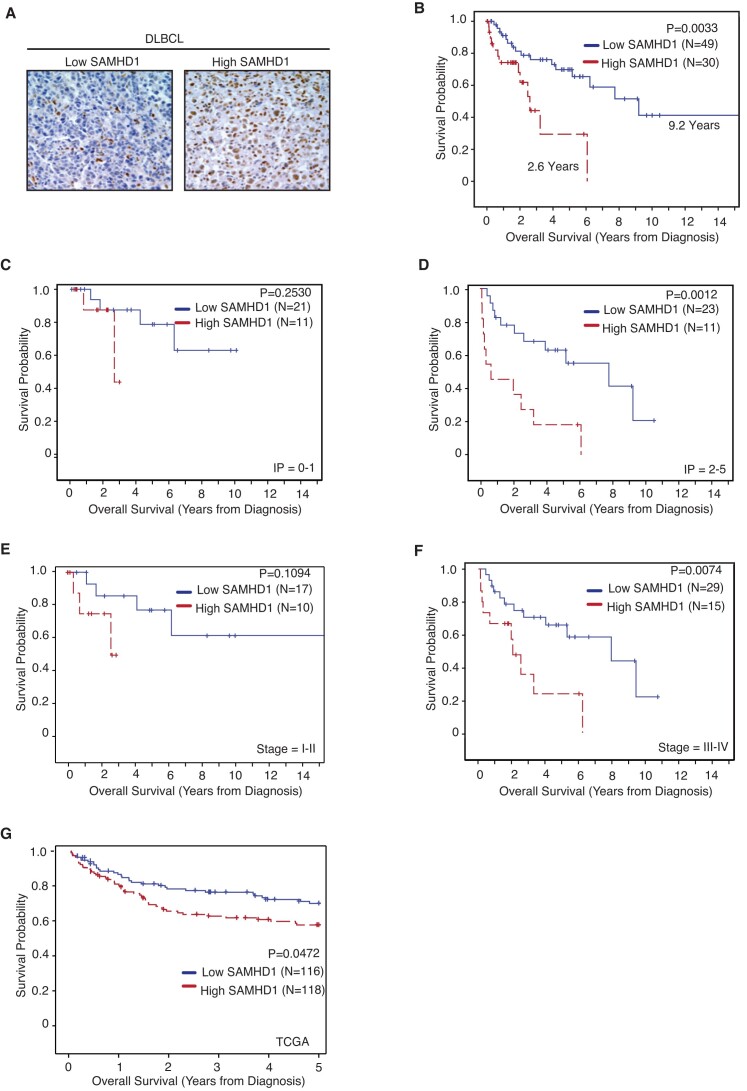
Low SAMHD1 expression is associated with improved overall survival in patients with DLBCL. (**A**) Representative immunohistochemical analysis for SAMHD1 expression in tissue from patients with DLBCL as used for scoring. (**B**) Kaplan–Meier plot showing patient outcome association with SAMHD1 expression level. Overall survival (OS) was defined as the time from diagnosis to death or the last contact when living patients were censored at the last contact. OS was estimated using the Kaplan–Meier method and was compared using log-rank tests. **(C–F)** The benefit of SAMHD1 expression level on OS of patients was assessed in a subgroup of patients with different IPI scores (C, D) and stages (E, F). (**G**) Analysis of an independent cohort of DLBCL patients from GDC indicates that low SAMHD1 expression is associated with improved OS.

To investigate the clinical relevance of our findings, we analyzed a database of 79 DLBCL patients treated at Emory University Hospital, for which treatment, survival, staging, and demographic data were available. The average percent SAMHD1 expression for all considered cases is presented in Supplementary Table S1, and the distribution of average SAMHD1 expression is in Supplementary Figure S3A and B. As shown in Table 1, the median age of the patients was 57.07 (range 20–86). 45 (57%) patients were 60 years or younger at diagnosis, and 34 (43%) were above 60. 27 (38%) patients were at stage I/II, and 44 (62%) were at stage III/IV, and 39 (50%) of the patients received R-CHOP, and 39 (50%) received other (defined as those who received CHOP, R-CHOP + additional treatment, or CHOP + additional treatment). 42 patients (63.6%) had LDH levels equal to or less than 270, and 24 patients (36.4%) had LDL levels above 270. IHC analysis of FFPE DLBCL specimens showed that 49 patients had low SAMHD1 expression and 30 patients had high SAMHD1 expression using >3, defined as the median as a cutoff (Table 1 and Figure 3A). On Kaplan–Meier log-rank analysis, patients with higher SAMHD1 expression demonstrated decreased overall survival (OS), with a median survival of 2.6 years (range 1.9 to 6.1) versus 9.2 years (range 5.2–16.6) for those with lower SAMHD1 expression; HR 3.16 and *P*= 0.003 (Figure 3B). On univariate Cox regression analysis, in addition to SAMHD1 expression, out of eight variables analyzed, age at diagnosis (*P*= 0.008), treatment (*P*= 0.002), LDH (*P*= 0.033) and IPI (*P*= 0.01) were also identified as a determinant of patient's OS (Table 2). On multivariate Cox regression analysis controlling for stage, treatment, age at diagnosis, and IPI, low SAMHD1 expression remained significantly associated with better OS (HR: 3.42 (95% CI: 1.49–7.83); *P*= 0.004) (Table 3), indicating that SAMHD1 expression is an independent OS determinant for DLBCL patients in this cohort. In the same analysis, treatment (HR: 0.26 (95% CI: 0.09–0.73); *P*= 0.011) and IPI (HR: 3.12 (95% CI: 1.25–7.77); *P*= 0.015) were also identified as independent determinants of OS. In the multivariate analysis, LDH was not considered because there were insufficient samples with the information. Consistent with our multivariate analysis, amongst DLBCL patients with high SAMHD1 expression, there was no statistically significant difference in clinicopathological variables (Table 4). Interestingly, on subgroup analysis, while low SAMHD1 expression did not show a significant benefit within low-risk DLBCL patients (IPI of 0–1; *P*= 02530), it was significantly beneficial in patients with moderate to high-risk disease (IPI = 2–5; *P*= 0.0012) (Figure 3C, D). In addition, DLBCL patients with stage III-IV DLBCL and low SAMHD1 levels showed significantly improved OS compared to those with high SAMHD1 expression (*P*= 0.0074). However, this benefit was insignificant in patients with stage I-II DLBCL (*P*= 0.1094) (Figure 3E, F). The findings in our subgroup analysis for patients with different IPI and stages suggest that low SAMHD1 expression may be more beneficial for those DLBCL patients with moderate to high risk (IPI 2–5) and more advanced stage (III–IV) for which about 60% of patients are diagnosed. As MYC/BCL2 double expression strongly predicts outcomes in patients with DLBCL treated with rituximab plus cyclophosphamide, doxorubicin, vincristine, and prednisone (51), we analyzed the correlation of expression of SAMHD1 with MYC and BCL2 but found no evidence of a significant correlation (*P*= 0.4) (Supplementary Figure S3C, D). To validate our finding of an association of low SAMHD1 expression with improved outcomes in DLBCL, we analyzed three publicly available datasets from the Genomics Data Commons (GDC) data portal (47), including 40 cases from The Cancer Genome Atlas (TCGA), 41 cases from Clinical Trial Sequencing Project (CTSP), and 481 cases from NCI Center for Cancer Research (NCICCR) with only the NCICCR dataset containing extractable survival data. In this dataset, low SAMHD1 expression correlated with a significantly improved 5-year OS (*P*= 0.0472) (Figure 3G). The distribution of SAMHD1 mRNA expression across the NCICCR patient dataset is presented in Supplementary Figure S3E. Collectively, these data suggest that low SAMHD1 expression is associated with improved OS in DLBCL patients.

**Table 1.** Descriptive Statistics for all patients (*N* = 79)

Variable	Level	*N*	Percent
SAMHD1 > 3	No	49	62.0
	Yes	30	38.0
Age at diagnosis	⇐60	45	57.0
	>60	34	43.0
Race	White	51	64.6
	Non-white	28	35.4
Sex	Female	36	45.6
	Male	43	54.4
Stage	I/II	27	38.0
	III/IV	44	62.0
	Missing	8	-
Treatment	Other	39	50.0
	R-CHOP	39	50.0
	Missing	1	-
LDH	⇐271	42	63.6
	>271	24	36.4
	Missing	13	-
IPI	0–1	32	48.5
	2–5	34	51.5
	Missing	13	-
Age at diagnosis	Mean	57.17	-
	Median	57.07	-
	Minimum	20.31	-
	Maximum	86.30	-
	Std Dev	14.43	-
	Missing	0	-

**Table 2.** Univariate Cox regression analysis for all patients (*N* = 79)

			Overall survival (years from diagnosis)		
Covariate	Level	*N*	Hazard Ratio (95% CI)	HR *P*-value	Log-rank *P*-value
SAMHD1 > 3	Yes	30	3.16 (1.41–7.06)	0.005	0.003
	No	49	-	-	
Age at diagnosis	>60	34	2.72 (1.26–5.86)	0.011	0.008
	⇐60	45	-	-	
Race	Non-white	28	1.12 (0.52–2.42)	0.773	0.773
	White	51	-	-	
Sex	Female	36	0.91 (0.44–1.91)	0.804	0.803
	Male	43	-	-	
Stage	III/IV	44	2.05 (0.87–4.82)	0.101	0.094
	I/II	27	-	-	
Treatment	R-CHOP	39	0.24 (0.09–0.63)	0.004	0.002
	Other	39	-	-	
LDH	>271	24	2.35 (1.05–5.26)	0.039	0.033
	⇐271	42	-	-	
IPI	2–5	34	3.10 (1.25–7.69)	0.015	0.010
	0–1	32	-	-	
Age at diagnosis		79	1.05 (1.02–1.09)	0.003	-

Abbreviations: HR, hazard ratio; R-CHOP, rituximab-cyclophosphamide, doxorubicin, vincristine, and prednisone; others, R-CHOP with radiation or additional chemo therapy. LDH, lactate dehydrogenase; IPI, international prognostic index.

**Table 3.** Multivariate Cox regression analysis for all patients (*N* = 79)

		Overall survival (years from diagnosis)	
Covariate	Level	Hazard Ratio	HR *P*-value
SAMHD1 > 3	Yes	3.42 (1.49–7.83)	**0.004**
	No	-	-
Stage	III/IV	1.98 (0.83–4.70)	0.122
	I/II	-	-
Treatment	R-CHOP	0.26 (0.09–0.73)	**0.011**
	Other	-	-
Age at diagnosis	>60	1.65 (0.71–3.83)	0.244
	⇐60	-	-
IPI	2–5	3.12 (1.25–7.77)	**0.015**
	0–1	-	-

*Number of observations in the original data set = 79. Number of observations used = 70.

Abbreviations: HR, hazard ratio; R-CHOP, rituximab-cyclophosphamide, doxorubicin, vincristine, and prednisone; others, R-CHOP with radiation or additional chemo therapy. IPI, international prognostic index.

**Table 4.** SAMHD1 statistics: comparisons for SAMHD1 > 3

Covariate	Statistics	Level	No *N* = 49	Yes *N* = 30	*P*-value*
Age at diagnosis	*N* (Row %)	⇐60	31 (68.89)	14 (31.11)	0.148
	*N* (Row %)	>60	18 (52.94)	16 (47.06)	
Race	*N* (Row %)	White	30 (58.82)	21 (41.18)	0.429
	*N* (Row %)	Non-white	19 (67.86)	9 (32.14)	
Sex	*N* (Row %)	Female	26 (72.22)	10 (27.78)	0.088
	*N* (Row %)	Male	23 (53.49)	20 (46.51)	
Stage	*N* (Row %)	I/II	17 (62.96)	10 (37.04)	0.801
	*N* (Row %)	III/IV	29 (65.91)	15 (34.09)	
Treatment	*N* (Row %)	Other	27 (69.23)	12 (30.77)	0.241
	*N* (Row %)	R-CHOP	22 (56.41)	17 (43.59)	
LDH	*N* (Row %)	⇐271	26 (61.9)	16 (38.1)	0.962
	*N* (Row %)	>271	15 (62.5)	9 (37.5)	
IPI	*N* (Row %)	0–1	21 (65.63)	11 (34.38)	0.862
	*N* (Row %)	2–5	23 (67.65)	11 (32.35)	

*The *P*-value is calculated by chi-square test or Fisher's exact, where appropriate.

## Discussion

This study demonstrates that low SAMHD1 expression or Vpx-mediated or siRNA-mediated degradation/depletion is associated with greater sensitivity to clinically relevant DSB-inducing agents doxorubicin and PARP inhibitor in DLBCL cells and that low SAMHD1 expression is associated with improved OS in DLBCL patients independent of other adverse factors, including IPI, and is most significant for those with advanced stage and higher risk. Thus, our data identify SAMHD1 expression as a biomarker of resistance to doxorubicin and PARP inhibitor in DLBCL cells and as a unique prognostic biomarker for outcome in patients with advanced stage higher risk DLBCL. In this regard, we found that SAMHD1 is differentially expressed in DLBCL cells and that low SAMHD1 expression or degradation with VLPs containing Vpx or siRNA-mediated depletion is associated with greater sensitivity to DSB-inducing agents doxorubicin and Veliparib, providing evidence that SAMHD1 mediates resistance to doxorubicin and Veliparib in DLBCL, and that the use of VLPs with Vpx to degrade SAMHD1 may be a potential therapeutic strategy to augment the efficacy of doxorubicin-based therapy in DLBCL.

Interestingly, SAMHD1 degradation with VLPs containing Vpx or siRNA-mediated depletion has a more significant effect in sensitizing SUDHL-4 and SUDHL-6 DLBCL cells with higher SAMHD1 expression than RCK-8 cells with lower SAMHD1 expression. SAMHD1 could be more critical for promoting doxorubicin resistance when expressed at higher levels. Thus, patients with high SAMHD1 expressing DLBCL may have the most potential for therapeutic gain using VLPs containing Vpx to degrade SAMHD1. The association of low SAMHD1 expression with Veliparib greater sensitivity in DLBCL cells is also consistent with a role for SAMHD1 in promoting the repair of DSBs by HR in DLBCL, indicating that its role in HR is also relevant in DLBCL. While R-CHOP is a multi-drug regimen, doxorubicin is the primary agent that induces DSBs (52,53). Thus, SAMHD1 may primarily be a biomarker for doxorubicin resistance in impacting clinical outcomes for DLBCL patients treated with R-CHOP although our model does not rule out the possibility that SAMHD1 may also mediate resistance to other R-CHOP agents that could indirectly induce DSBs.

Our clinical data show that low SAMHD1 expression is associated with improved OS in patients with DLBCL. This was confirmed in an independent cohort of DLBCL patients using GDC data and in multivariate analysis, implying that SAMHD1 is an independent prognostic biomarker for outcomes in DLBCL patients. Given that most patients in our study received doxorubicin-based therapy and that low SAMHD1 expression is associated with doxorubicin greater sensitivity, SAMHD1 expression may also have predictive significance for patients who receive doxorubicin-based treatment. However, this requires further evaluation, including a larger data set with an untreated cohort and/or cohort treated with non-doxorubicin-based therapy. Our subgroup analysis for patients with different IPI and stages suggests that low SAMHD1 expression may benefit DLBCL patients with moderate to high risk (IPI 2–5) and more advanced stages (III-IV) for which about 60% of patients are diagnosed. However, a trend for an association of low SAMHD1 expression with improved OS was also noted for patients with low risk and early stage. This could be attributed to this subset's lower number of death events during our follow-up period.

Viral vectors have been explored as a formidable gene and protein delivery system for tumor or cancer cells and several advanced clinical trials (57–59). Vpx has been explored as a potential therapeutic tool for hematological malignancies, and VLPs are safer viral delivery systems being investigated for diverse clinical applications ((26,56) and reviewed in (60)). Given our data showing that low SAMHD1 expression is associated with improved outcomes for DLBCL patients, it is possible that treatment regimens could be tailored to individualize DLBCL patient treatment based on SAMHD1 expression. In particular, our data suggest that even amongst patients with a specific IPI risk group, there are subsets of patients with better or worse outcomes. Thus, patients with high SAMHD1 expression may benefit from more aggressive treatment regimens and/or participation in clinical trials. Limitations of our study include relatively small patient numbers and inherent biases in a retrospective study design. In addition, the genomic data analysis was performed on tissue biopsies, and no information was provided in the dataset reference as to whether infiltrating reactive T-cells and macrophages were accounted for, which could be a confounding variable. Ultimately, the clinical utility of SAMHD1 expression as a prognostic or potentially predictive biomarker requires further validation in a prospective clinical trial.

## Supplementary Material

zcae007_supplemental_file

## Data Availability

The data underlying this article will be shared on reasonable request to the corresponding author.
