# Supplementary material for: SAMHD1 expression contributes to doxorubicin resistance and predicts survival outcomes in diffuse large B-cell lymphoma patients
Source: NAR Cancer. 2024 Feb 24;6(1):zcae007. doi: 10.1093/narcan/zcae007 (PMC10894040; doi:10.1093/narcan/zcae007)
Supplement: zcae007_supplemental_file [file zcae007_supplemental_file.pdf]

Supplementary materials

SAMHD1 expression contributes to doxorubicin resistance and predicts survival outcomes in diffuse large B-cell lymphoma patients

Waaqo Daddacha[6*], Dominique Monroe[6], Ashley J. Schlafstein[1], Allison E. Withers[1], Elizabeth B. Thompson[1], Diana Danelia[1], Nho C. Luong[1], Fatmata Sesay[1], Sandip K. Rath[1], Edidiong R. Usoru[6], Mark E. Essien[1], Andrew T. Jung[1], Jinmeng G. Jiang[1], Jiaxuan Hu[1], Bijan Mahboubi[2], Ariyln Williams[6], Julia E. Steinbeck[6], Xiaofeng Yang[1], Zachary S. Buchwald[1], William S. Dynan[1,3], Jeffrey M. Switchenko[4], Baek Kim[2], Mohammad Khan[1], David L. Jaye[5], and David S. Yu[1,*]

[1] Department of Radiation Oncology, Emory University School of Medicine, Atlanta, GA 30322 USA, [2] Department of Pediatrics, Emory University School of Medicine, Atlanta, GA 30322, [3] Department of Biochemistry, Emory University School of Medicine, Atlanta, GA 30322, USA Department of Biochemistry, Emory University School of Medicine, Atlanta, GA 30322, USA, [4] Department of Biostatistics and Bioinformatics, Rollins School of Public Health and Winship Cancer Institute, Emory University School of Medicine, Atlanta, GA 30322, USA, [5] Department of Pathology and Laboratory Medicine, Emory University School of Medicine, Atlanta, GA 30322, [6] Department of Biochemistry and Molecular Biology, Medical College of Georgia, Augusta University, Augusta, GA 30912

[*] Corresponding Authors:
David S. Yu, M.D., Ph.D.
Department of Radiation Oncology
Emory University School of Medicine
1365 Clifton Rd NE, C3008
Atlanta, GA 30322
Phone: 404-778-1758, Fax: 404-778-5520
e-mail: dsyu@emory.edu

Waaaqo Daddacha, Ph.D.
Department of Biochemistry and Molecular Biology
Augusta University, Medical College of Georgia
1410 Laney Walker Blvd., CN2176
Augusta, GA 30912
Phone: 706-721-0272
e-mail: wdaddacha@augusta.edu

A

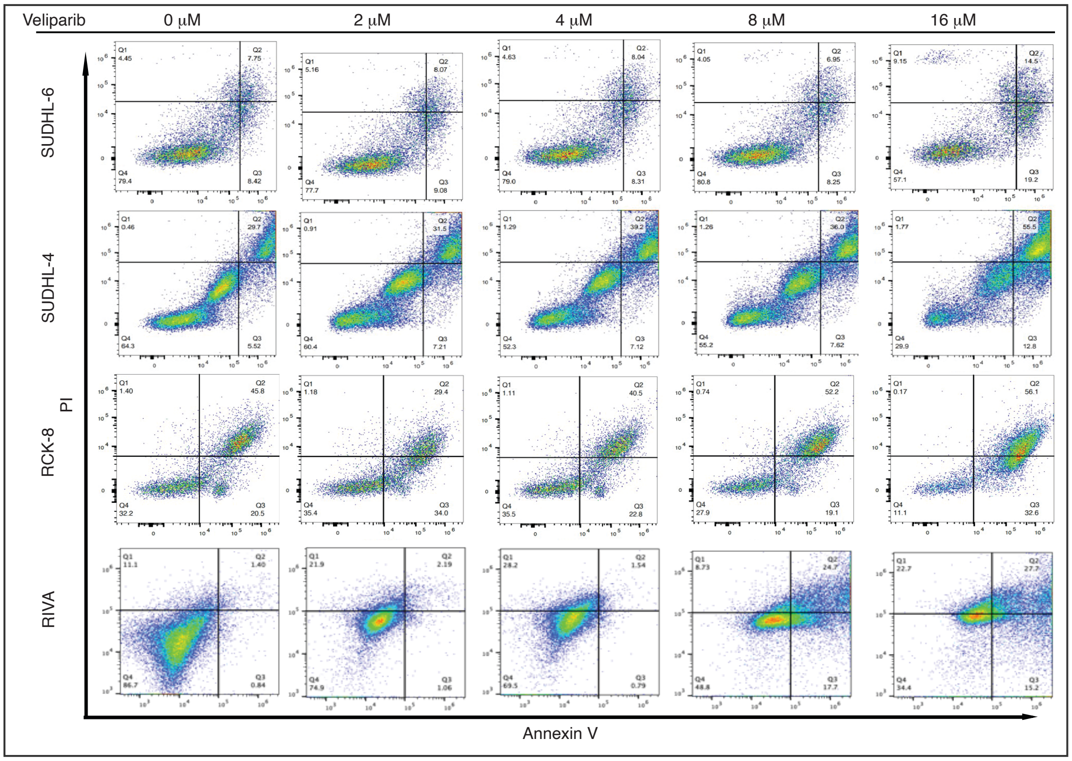

Veliparib
0 μM
2 μM
4 μM
8 μM
16 μM
SUDHL-6
SUDHL-4
RCK-8
RIVA
PI
Annexin V

B

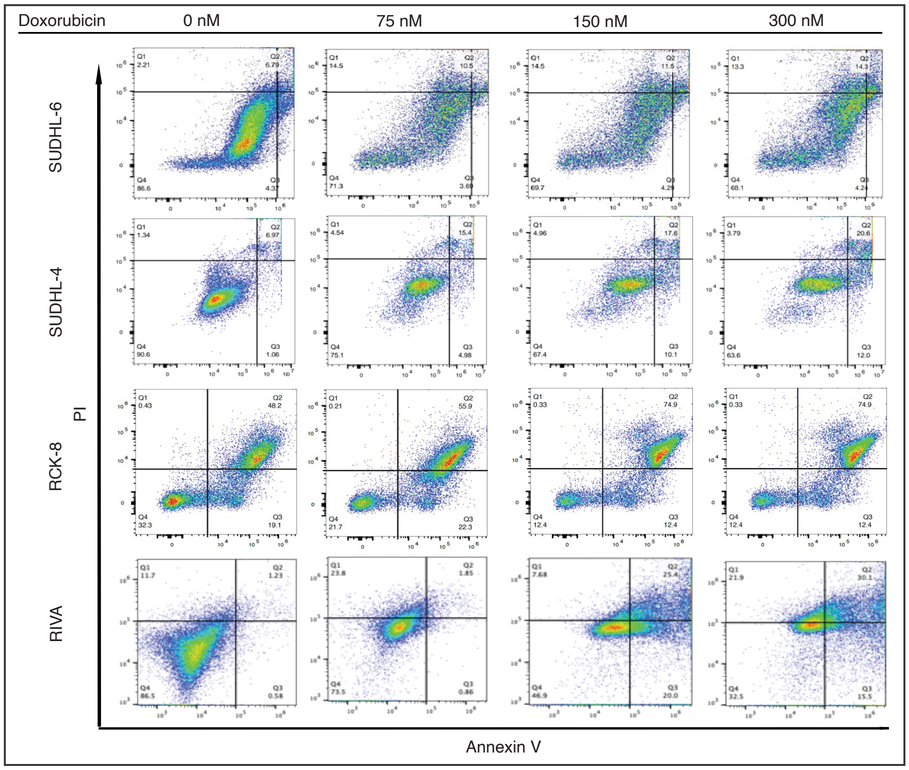

Doxorubicin
0 nM
75 nM
150 nM
300 nM
SUDHL-6
SUDHL-4
RCK-8
RIVA
PI
Annexin V

Supplementary Figure S1. Evaluation of cell viability using Annexin V and Propidium Iodide (FITC/PI) staining following treatment of cells with **(A)** 0, 2, 4, 8, and 16 uM Veliparib or **(B)** 0, 75, 150, 300 nM Doxorubicin. Population in quadrant 4 were selected and considered viable cells. Representative flow cytometry data from 3 replicas are shown.

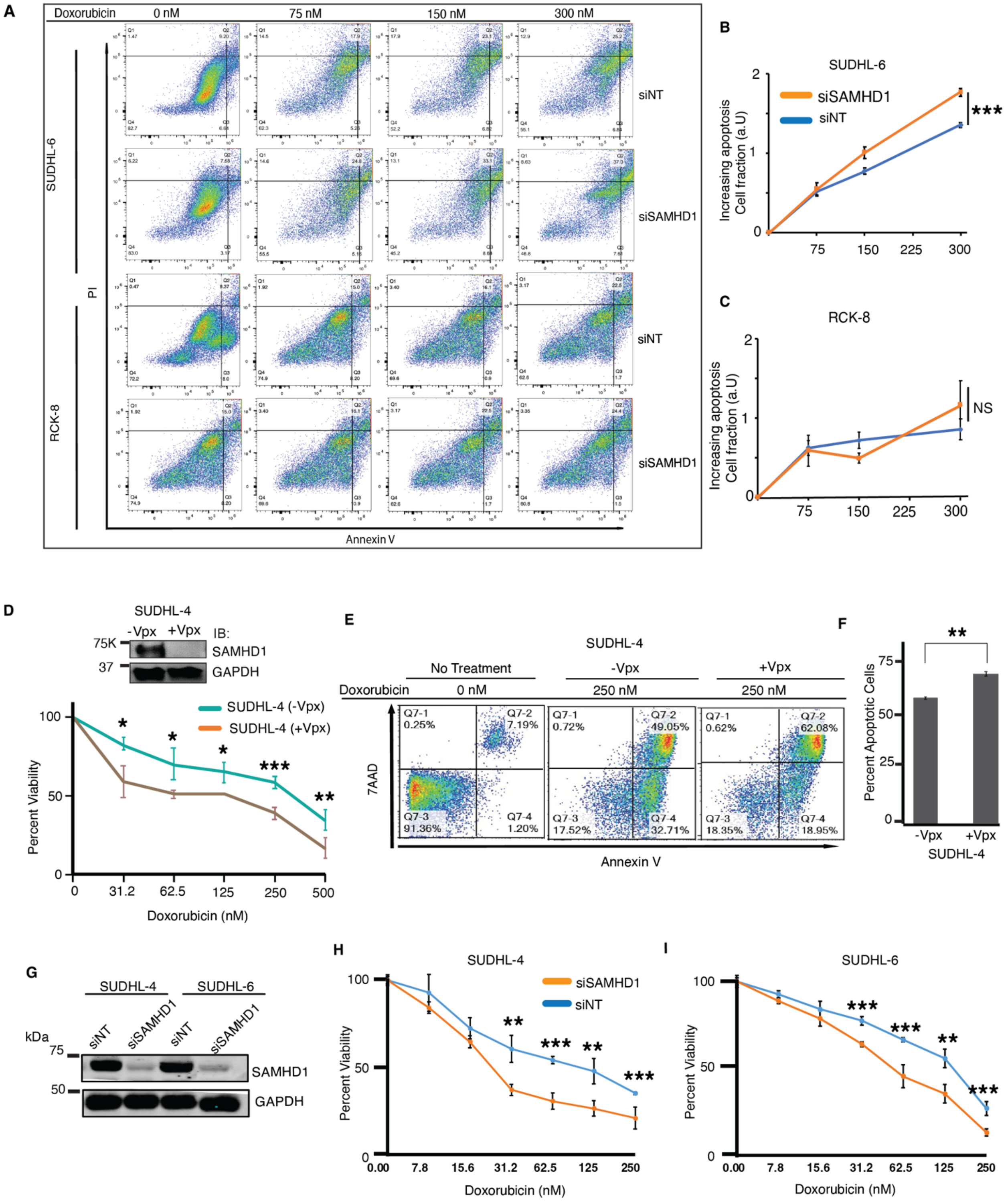

A
Doxorubicin
0 nM
75 nM
150 nM
300 nM
SUDHL-6
RCK-8
PI
siNT
siSAMHD1
siNT
siSAMHD1
Annexin V
B
SUDHL-6
siSAMHD1
siNT
***
Increasing apoptosis
Cell fraction (a.U)
0
1
2
75
150
225
300
C
RCK-8
NS
Increasing apoptosis
Cell fraction (a.U)
0
1
2
75
150
225
300
D
SUDHL-4
-Vpx
+Vpx
IB:
75K
SAMHD1
37
GAPDH
SUDHL-4 (-Vpx)
SUDHL-4 (+Vpx)
Percent Viability
0
50
100
*
*
*
***
**
0
31.2
62.5
125
250
500
Doxorubicin (nM)
E
SUDHL-4
No Treatment
-Vpx
+Vpx
Doxorubicin
0 nM
250 nM
250 nM
7AAD
Q7-1
0.25%
Q7-2
7.19%
Q7-3
91.36%
Q7-4
1.20%
Q7-1
0.72%
Q7-2
49.05%
Q7-3
17.52%
Q7-4
32.71%
Q7-1
0.62%
Q7-2
62.08%
Q7-3
18.35%
Q7-4
18.95%
Annexin V
F
**
Percent Apoptotic Cells
0
25
50
75
-Vpx
+Vpx
SUDHL-4
G
SUDHL-4
SUDHL-6
kDa
siNT
siSAMHD1
siNT
siSAMHD1
75
50
SAMHD1
GAPDH
H
SUDHL-4
siSAMHD1
siNT
Percent Viability
0
50
100
**
***
**
***
0.00
7.8
15.6
31.2
62.5
125
250
Doxorubicin (nM)
I
SUDHL-6
Percent Viability
0
50
100
***
***
**
***
0.00
7.8
15.6
31.2
62.5
125
250
Doxorubicin (nM)

Supplementary Figure S2. (A) Evaluation of SUDHL-4 and SUDHL-6 viability using Annexin V and Propidium Iodide (FITC/PI) staining following transfection with siNT and siSAMHD1 and treatment with 0, 75, 150 and 300 nM Doxorubicin. Population in Quadrant 4 were selected and considered viable cells. In addition, the apoptotic population was analyzed and plotted (B-C). **(D)** SUDHL-4 cells were seeded in 96 well plates, transduced with VLP containing Vpx, treated with the indicated concentration of Doxorubicin, and sensitivity analyzed via AlamarBlue-based cell viability assay. **(E-F)** SUDHL-4 sensitivity was determined via Annexin V/7AAD-based assay following transduction with VLP containing Vpx and treatment with 250 nM Doxorubicin. Shown are the flow data **(E)** and the quantification **(F). (G-I)** SUDHL-4 and SUDHL-6 cells transfected with siSAMHD1, treated with varying concentrations of doxorubicin, and sensitivity analyzed via AlamarBlue-based assay. Shown are western blot demonstrating SAMHD1 KD **(G)** and quantified and plotted data **(H -I).** For **B-D** and **H-I,** the mean and standard deviation obtained from 3 replicas are shown. *** p<001, ** p < 0.01, * p<0.05.

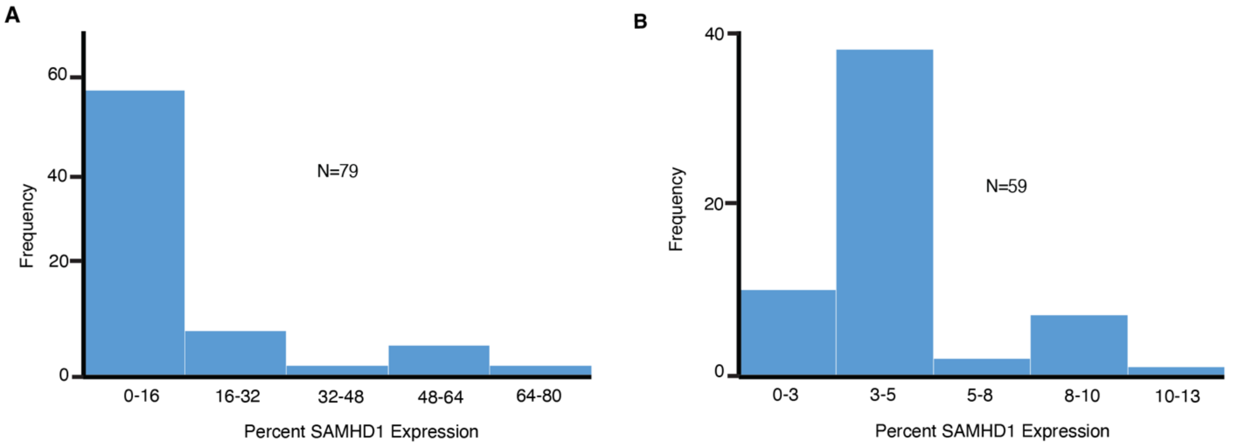

C

	Percent SAMHD1 Average	Standard Deviation	Number of Samples
**Double Positive MYC and BCL2**	**20**	**26**	**13**
**Double Negative MYC and BCL2**	**11**	**17**	**47**
**p=0.4**			

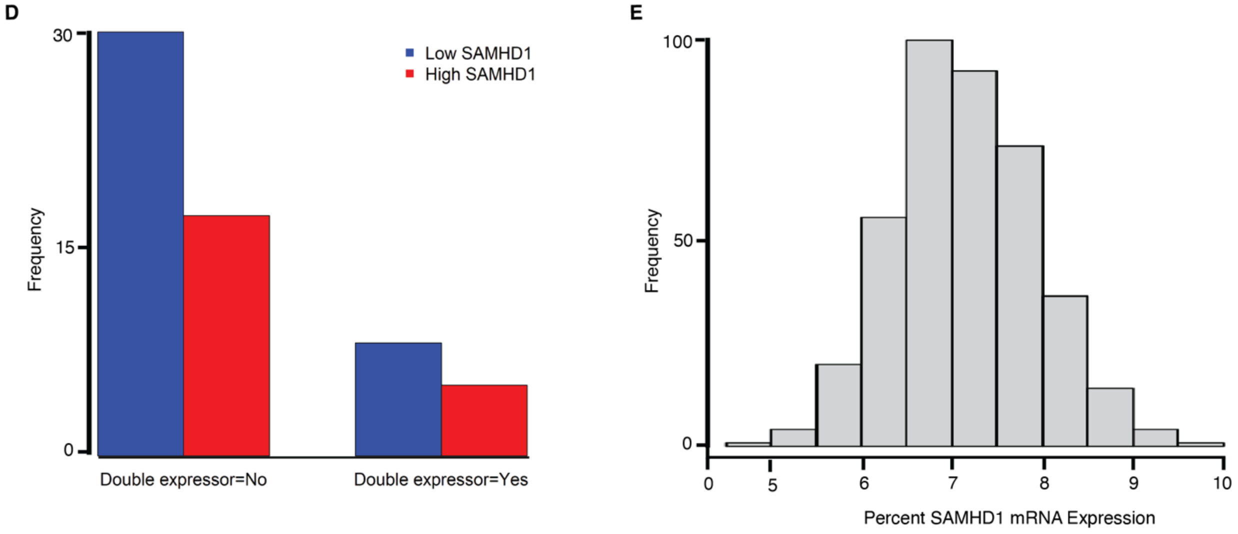

Supplementary Figure S3. Samples of 79 DLBCL patients treated at Emory University Hospital and with survival information were analyzed for SAMHD1 expression. The distribution was plotted for **(A)** all samples and **(B)** 59 samples in the majority (0-13) percent expression. **(C)** Out of the 79 samples, those with MYC and BCL2 double expression were analyzed for correlation with SAMHD1 expression. **(D)** Frequency of MYC and BCL2 double expression dichotomized with high or low SAMHD1 expression. **(E)** The patient cohort from NCICCR dataset (N=234), which has survival information, was analyzed for SAMHD1 mRNA expression and the distribution potted.

Supplementary Table 1. Percent Total Tumor Cell Expression: SAMHD1, MYC, and BCL2. MYC/BLC2 double expressing was defined as >=40% MYC and >=50% BCL2.

TMA	MYC Avg	SAMHD1 Average	BCL2 avg	Double Expressors
2-13	0	0	25	0
2-16	0	0	30	0
2-18	0	0		0
2-23	28	0	33	0
2-28	10	0		0
3-19	0	0	13	0
3-9	0	2	0	0
3-1	8	3	18	0
1-2	38	3	90	0
1-7	5	3	80	0
1-9	30	3	96	0
1-10	18	3	2	0
1-12	8	3	83	0
1-13	10	3	25	0
1-14	5	3	0	0
1-18	5	3	16	0
1-19	0	3	68	0
1-26	20	3	96	0
1-27	33	3	98	0
2-2	13	3	93	0
2-3	0	3	1	0
2-6	5	3	35	0
2-15	90	3	3	0
3-6	0	3	85	0
3-7	10	3	80	0
3-11	35	3	45	0
3-12	25	3	90	0
3-17	25	3	95	0
3-18	20	3	25	0
3-22	18	3	18	0
3-20	6	5	96	0
3-10	28	5	90	0
2-20		5		0
1-23	18	8	99	0
2-1	38	8	23	0
2-29	10	8	63	0
1-11	60	20	8	0
3-8	23	20	80	0
3-4	25	23	0	0

1-28	25	23	83	0
2-26	20	30	75	0
2-7	30	33	10	0
1-25	30	55	80	0
2-12	5	60	98	0
1-8	30	63	93	0
1-21	80	63	2	0
2-27	0	77		0
3-29	80	0	98	1
1-5	53	3	98	1
1-29	53	3	75	1
2-11	68	3	90	1
3-2	83	3	96	1
3-3	55	3	93	1
3-13	33	3	85	1
3-14	30	3	88	1
1-4	63	10	85	1
1-24	45	43	95	1
1-20	80	50	93	1
2-30	75	60	93	1
1-6	48	70	63	1
2-24		0		
3-24		0		
3-26		0		
1-15		1		
1-17		3		
1-1	-	3	-	
1-16		3		
2-4		3		
2-10		3		
3-15		3		
3-16		3		
3-28		3		
3-25		5		
3-21		10		
2-21		10		
3-5		10		
1-22		12		
2-25		27		
2-5		30		
2-8		30		
2-9		30		
3-23		30		

3-27		80		
